# Associations between vitamin D status and biomarkers linked with inflammation in patients with asthma: a systematic review and meta-analysis of interventional and observational studies

**DOI:** 10.1186/s12931-024-02967-z

**Published:** 2024-09-19

**Authors:** Asmae El Abd, Harika Dasari, Philippe Dodin, Helen Trottier, Francine M. Ducharme

**Affiliations:** 1 Sainte-Justine University Health Center, Research Center, Montreal, Quebec Canada; 2 Department of Social and Preventive Medicine, School of Public Health, University of Montreal, Montreal, Quebec Canada; 3 Department of Pediatrics, Faculty of Medicine, University of Montreal, Sainte-Justine Hospital, Montreal, Quebec Canada

**Keywords:** Inflammation, Asthma, Vitamin D, Biomarkers, Systematic review, Meta-analysis

## Abstract

**Background:**

Numerous studies indicate an association between vitamin D status and inflammatory biomarkers in patients with asthma, but findings are inconsistent. This review aims to summarize the relationship between serum vitamin D status, assessed by 25-hydroxyvitamin D (25(OH)D) level, and inflammatory biomarkers in children and adults with asthma.

**Methods:**

A literature search of interventional and observational studies on 25(OH)D up to November 2022 was conducted across six electronic databases. Outcomes of interest included a range of inflammatory biomarkers classified in four categories: T helper 2 (Th2) pro-inflammatory, non-Th2 pro-inflammatory, anti-inflammatory, and non-specific biomarkers. Study characteristics were extracted and risk of bias was evaluated using the American Academy of Nutrition and Dietetics tool. Meta-analysis was conducted on studies with a low risk of bias, while narrative reporting was used to present the direction of associations (positive, no association, or negative) for each biomarker, overall and within the low-risk studies.

**Results:**

We included 71 studies (3 interventional, 68 observational) involving asthma patients. These studies investigated the association between serum 25(OH)D and Th2 pro-inflammatory biomarkers (*N* = 58), non-Th2 pro-inflammatory biomarkers (*N* = 18), anti-inflammatory biomarkers (*N* = 16), and non-specific biomarkers (*N* = 10). Thirteen (18.3%) studies, 50 (70.4%), and 8 (11.3%) were at high, moderate, and low risk of bias, respectively. In all studies, irrespective of risk of bias, the most frequently reported finding was no significant association, followed by a negative association between 25(OH)D and pro-inflammatory biomarkers and a positive association with anti-inflammatory biomarkers. In low-risk studies, one biomarker could be meta-analysed. The pooled estimate for 25(OH)D and serum IgE showed a negative association (β (95% CI)= − 0.33 (–0.65 to − 0.01); I^2^ = 88%; *N* = 4 studies). A negative association between 25(OH)D and blood eosinophils was also observed in the largest of three studies, as well as with cathelicidin (LL-37) in the only study reporting it. For other biomarkers, most low-risk studies revealed no significant association with 25(OH)D.

**Conclusion:**

Serum 25(OH)D is negatively associated with serum IgE and possibly with blood eosinophils and LL-37, supporting an in vivo immunomodulatory effect of 25(OH)D. Future research should employ rigorous methodologies and standardized reporting for meta-analysis aggregation to further elucidate these associations.

**Supplementary Information:**

The online version contains supplementary material available at 10.1186/s12931-024-02967-z.

## Introduction

Asthma is a chronic respiratory disease characterized by bronchial hyperreactivity and inflammation leading to preventable symptoms and exacerbations [1, 2]. In 2019, approximately 262 million people were affected by asthma, resulting in 455,000 deaths [3]. Asthma is characterized by chronic inflammation that is mediated, in many patients, by T helper 2 (Th2) cytokines, which can enhance the infiltration of inflammatory cells into the airways, activate resident immune cells, and trigger the release of inflammatory mediators [4]. Inflammatory biomarkers measured in sputum and serum can be used in disease management, prognosis, and therapeutic adjustment [5]. Key biomarkers include pro-inflammatory markers of Th2 inflammation, often measured in clinical practice and research in asthma patients, such as immunoglobulin E (IgE) [5–7], eosinophils [5, 8, 9], fractional exhaled nitric oxide (FeNO) [5, 10], interleukin (IL)-4, and IL-5 [11], as well as non-Th2 pro-inflammatory biomarkers, including neutrophils [12, 13], C-reactive protein (CRP) [14, 15], tumor necrosis factor α (TNF-α) [16], and interferon gamma (IFN-γ) [17]. Additionally, several anti-inflammatory biomarkers, including regulatory T cells (Tregs) [18, 19], the antimicrobial peptide cathelicidin (LL-37) [20], and IL-10 [11], have also been studied in patients with asthma.

Vitamin D deficiency and insufficiency represent a global public health problem affecting individuals of all ages, with more than one billion children and adults at risk worldwide [21, 22]. The Institute of Medicine (IOM) classifies vitamin D deficiency and insufficiency as a serum 25-hydroxyvitamin D (25(OH)D) concentration less than 30 nmol/L and less than 50 nmol/L, respectively [23]. In patients with asthma, a lower level of serum 25(OH)D has been associated with a greater risk of hospital admissions, emergency department visits, exacerbations, and use of rescue oral corticosteroids (OCS) for asthma [24, 25]. Several extraosseous anti-inflammatory and immunomodulatory effects of vitamin D have been demonstrated through in vivo studies using animal models, as well as through in vitro cell cultures [26]. Perhaps even more convincing is the evidence from meta-analyses of randomized controlled trials (RCTs), which report an increase in the levels of anti-inflammatory cytokines, such as IL-10, in both children and adults with asthma after vitamin D supplementation with doses ranging from 800 to 400,000 IU over periods of 6 weeks to 12 months [27]. However, the relationship between inflammatory biomarkers and vitamin D status, as assessed by 25(OH)D—the most reliable indicator of vitamin D levels in the human body, influenced not only by vitamin D supplementation but also by factors such as diet, sunlight exposure, and genetic predisposition [28]—is not yet clear. Indeed, numerous observational epidemiological studies conducted in children and adults with asthma have reported an inverse association between serum 25(OH)D levels and pro-inflammatory Th2 [29–36] and non-Th2 [36–40] biomarkers, as well as a positive association with anti-inflammatory biomarkers [40, 41]. However, there is significant heterogeneity and inconsistency in these findings. Notably, some studies suggest a different direction of the association between 25(OH)D and these inflammatory biomarkers in asthma [36, 42–44], while others have failed to demonstrate any significant association at all [29, 36, 39, 45–50]. Given these discrepancies, a comprehensive review is crucial.

This systematic review aimed to synthesize the current evidence on the association between vitamin D status (25(OH)D) and inflammatory biomarkers in children and adults with asthma.

## Materials and methods

The research protocol (#CRD42022365666) was registered in the International Prospective Register of Systematic Reviews (PROSPERO). We conducted this systematic review and meta-analysis in accordance with guidelines specified by the Preferred Reporting Items for Systematic Review and Meta-analysis (PRISMA) statement [51].

### Search strategy

A comprehensive search for relevant articles published up to November 24, 2022, was conducted by a trained librarian (PD) in six databases, namely PubMed, Medline, All EBM (evidence-based medicine), Embase, CINAHL (cumulative index to nursing and allied health literature), and Web of Science. The complete search strategy, including Medical Subject Headings (MESH) terms and keywords used for each database, is detailed in Additional file 1: Method S1. We also conducted a manual search of the bibliography of the selected articles to identify additional relevant studies.

### Selection criteria

The population of interest consists of individuals diagnosed with asthma of any age (children and adults), sex, or ethnicity. Studies were eligible if they were RCTs, pre-post intervention studies, or observational studies (cohorts, case‒control, or cross-sectional designs) investigating the association between vitamin D status (25(OH)D) and inflammatory biomarkers in patients with asthma. The exposure of interest was vitamin D status, either reported as (i) continuous 25(OH)D serum levels or (ii) categorical variable based on definitions (e.g., deficiency, insufficiency, or sufficiency) used by included studies. Outcomes of interest were all inflammatory biomarkers that were reported in included studies, grouped as follows: (i) pro-inflammatory biomarkers of Th2 inflammation (i.e., IgE, eosinophils, FeNO, Eosinophil Cationic Protein [ECP], IL-4, IL-5, IL-13, and IL-33); (ii) pro-inflammatory biomarkers of non-Th2 inflammation (i.e., IL-1, IL-2, IL-3, IL-6, IL-8, IL-9, IL-12, IL-17, IL-31, CRP, Hs-CRP [high-sensitivity C-reactive protein], TNF-α, IFN-γ, interferon gamma-induced protein 10 (IP-10), neutrophils, and polymorphonuclear [PMN] cells); (iii) anti-inflammatory biomarkers (i.e., Tregs, IL-10, LL-37, transforming growth factor-beta 1 [TGF-β1], and adiponectin); and (iv) non-specific biomarkers (i.e., leucocytes, lymphocytes, platelets, Th1/Th2 ratio, hemoglobin, IgG, IgM, IgA, leptin, and resistin).

We did not consider the following designs: animal models, in vitro studies, narrative and systematic reviews, opinion papers, case reports, or abstract and conference papers. We also excluded studies involving mother‒child pairs where vitamin D levels were measured in either the mother or the umbilical cord blood at or before delivery and where inflammatory biomarkers were assessed in the children. Additionally, we did not include interventional studies where patients received any kind of supplementation other than vitamin D (e.g., anti-IgE therapy) or were exposed to vitamin D alongside other vitamins or mineral supplements, except for standard asthma therapy. This exclusion criterion was applicable to interventional studies in which the inflammatory biomarker was not assessed at baseline before supplementation, but only at the endpoint or as a change from baseline. This approach was taken to avoid confounding effects of other immunomodulatory interventions on inflammatory biomarkers and to ensure that our analysis focused solely on the relationship between 25(OH)D and inflammatory biomarkers.

### Study selection and data extraction

Two reviewers (AEA and HD) independently screened all titles and abstracts and then reviewed the full texts of those studies deemed relevant. The following information was extracted independently using a predesigned form: study design, location, sample size, study population characteristics (number, age, and % male), vitamin D status, inflammatory biomarker results, main statistical analysis methods, and, if applicable, adjustment variables included in statistical models. When multivariable models were presented, we extracted the results from the model with the best fit, including adjustment for relevant variables. All conflicts between reviewers related to study selection and data extraction were resolved by discussion; the input of a third independent reviewer (FMD) was solicited in cases of disagreement. Covidence systematic review software (2023, Veritas Health Innovation, Melbourne, Australia) was used to manage and streamline the process.

### Quality assessment

Two independent reviewers (AEA and HD) evaluated the methodological quality of included studies using the standardized critical appraisal checklist designed by the American Academy of Nutrition and Dietetics [52] which comprises ten validity questions related to study design, addressing aspects such as the research question, study population, sampling, intervention or exposure, outcome measurements, statistical analysis, and interpretation of findings. This tool offers a meticulous and rigorous approach to ensure objectivity, transparency, and reproducibility, demonstrating higher inter-observer agreement compared to the Cochrane risk-of-bias tool [53]. It is also specifically adapted to nutrition-related topics, with a comprehensive focus on nutritional interventions and exposures, making it particularly useful and relevant for our research question [54]. Reviewers assessed each study individually and determined its quality as high, moderate (neutral), or low risk of bias. Any disagreement regarding the methodological quality of a study was resolved by reaching a consensus or by consulting a third reviewer (FMD).

### Qualitative and quantitative analysis

The results were presented both narratively and in tabulated form. A meta-analysis was planned only in studies that used multivariate analysis to adjust for potential confounding factors or imbalances between groups, when the following criteria were met: (i) at least two studies measured the same inflammatory biomarker from the same body fluid using identical parametrization (i.e., continuous or categorical variable); (ii) adjusted β coefficients from multivariable linear regression and standard errors (SE) or 95% confidence intervals (CI) reported for inflammatory biomarkers analysed as a continuous variable; (iii) adjusted odds ratios (OR) from multivariable logistic regression and SEs or 95% CIs reported for inflammatory biomarkers analysed as a categorical variable; and (iv) rated as being at low risk of bias. For inflammatory biomarkers measured at different time points, we only considered the baseline time point. All 95% confidence intervals (CIs) were converted in SEs to facilitate the calculation of the pooled associations. Heterogeneity was assumed if I² was greater than 50% and the P value was less than 0.1 [55]. Adjusted estimates were combined using the inverse variance method with fixed-effects model when I² was less than 50%; else, a random-effects model was used. To explore the source of heterogeneity, we planned to conduct subgroup analyses on study design (cohort, case‒control, vs. cross-sectional studies), patient age (children vs. adults), and baseline 25(OH)D levels (< 50 nmol/L vs. ≥ 50 nmol/L). A sensitivity analysis was planned after excluding studies with asymmetric data distribution, where the conversion of the 95% CI into SE was imprecise. The analysis was performed using R software, version 2023.09.1 + 494.

When meta-analyses for a given biomarker could not be conducted (i.e., not meeting our stated criteria for aggregation), the following complementary approach was used: for each inflammatory biomarker, we counted the number of studies reporting a significant negative, positive, or no statistically significant association, considering the results from all studies, irrespective of the risk of bias, and then focusing only on studies at low risk of bias.

## Results

### Search results

**Fig. 1:**
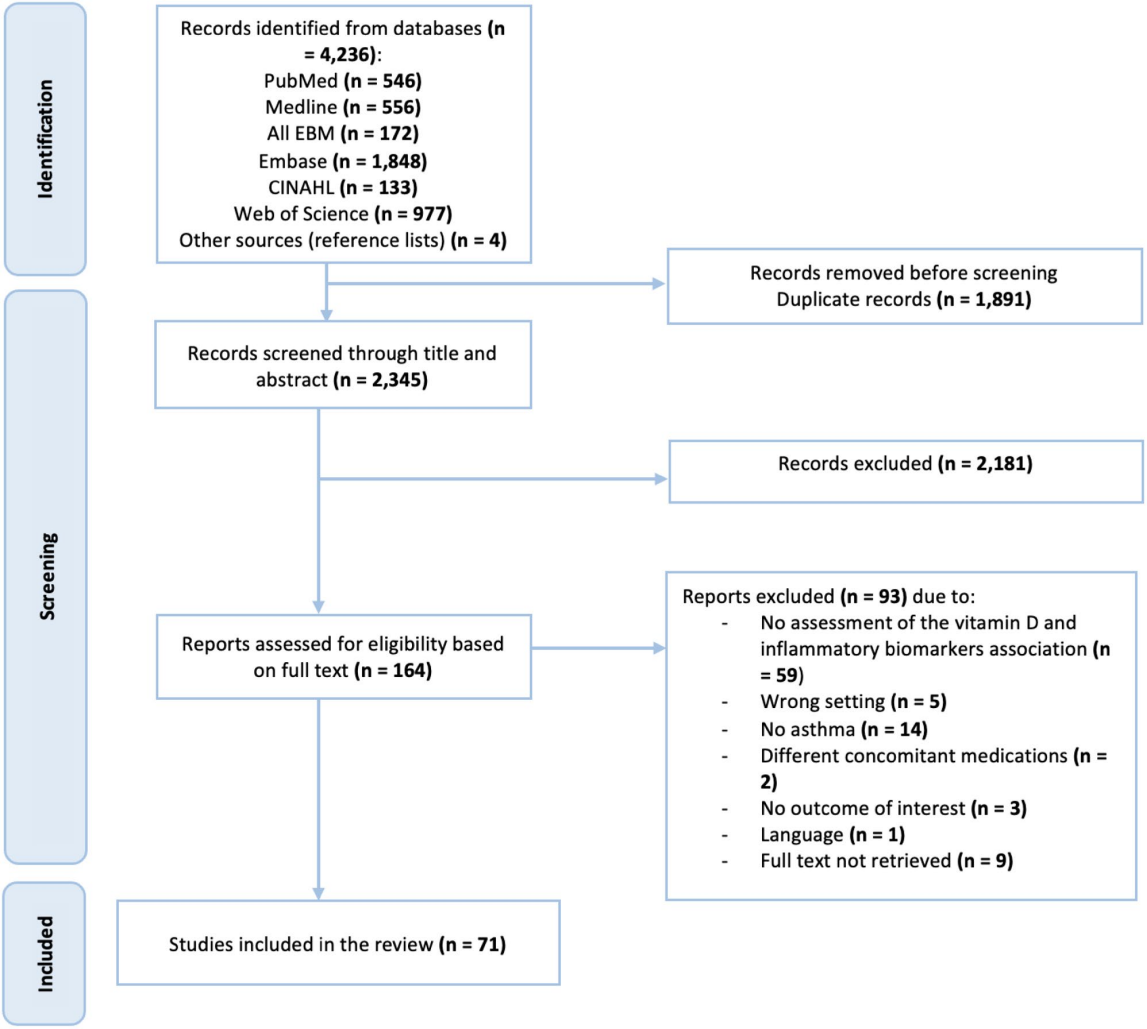
Selection process for eligible studies from all identified citations

The literature search yielded a total of 4,236 publications. After removing 1,891 duplicate records, the remaining 2,345 citations were screened by their title and abstracts, 164 articles were selected for full-text assessment. Subsequently, 93 studies were excluded for various reasons, such as not assessing the association between 25(OH)D and inflammatory biomarkers (59 studies), not focusing on asthma (14 studies), and lacking relevant outcomes (3 studies). Finally, 71 studies were included in the review (Fig. 1).

### Study characteristics

**Fig. 2:**
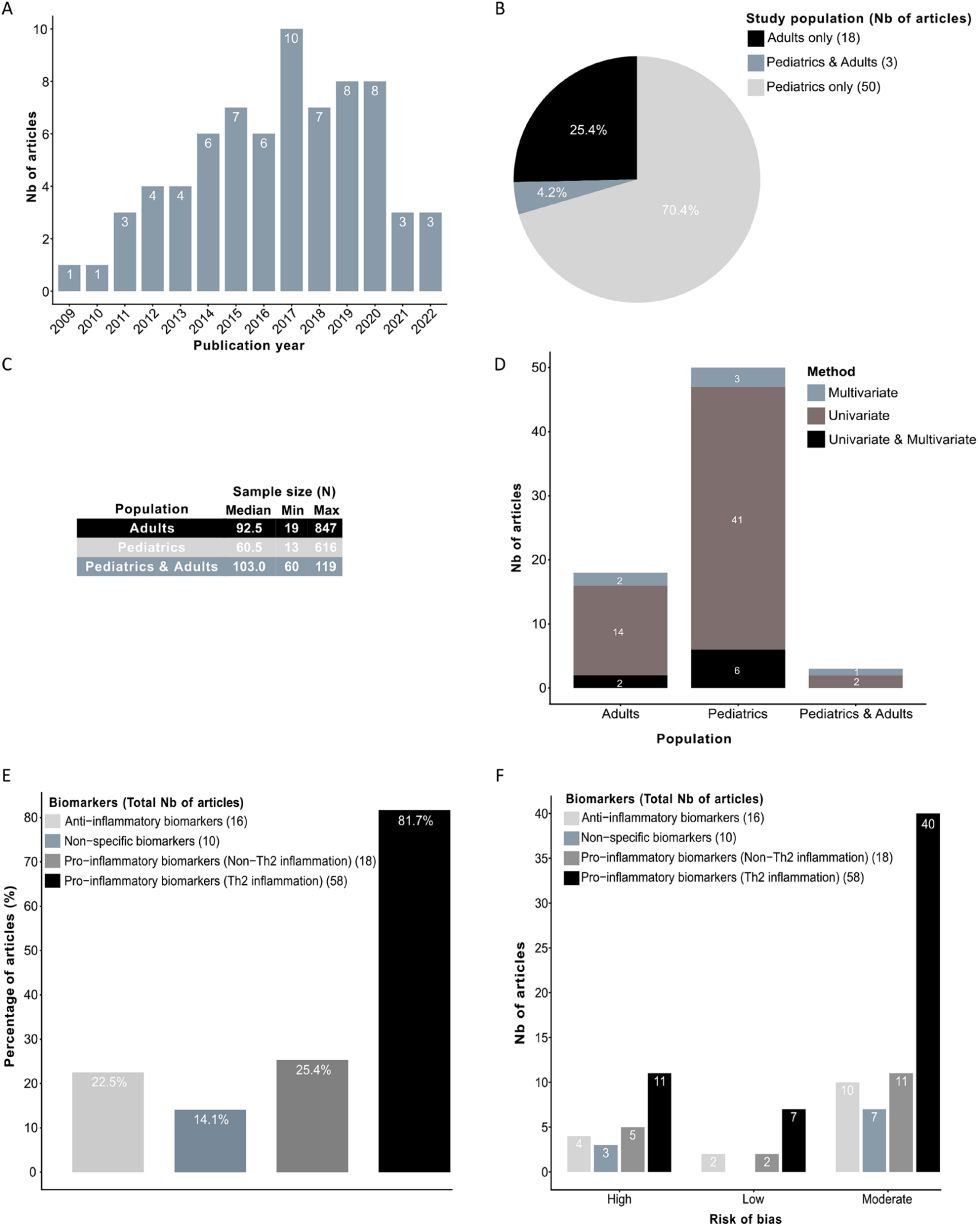
Overview of some study characteristics of the studies included in the systematic review. (**A**) Number of studies based on the year of publication; (**B**) Percentage (count) of studies by population type; (**C**) Descriptive statistics on sample size grouped by type of population; (**D**) Number of studies according to the type of statistical analysis carried out and the type of population; (**E**) Number of studies according to type of biomarkers. (**F**) Percentage (count) of studies reporting at least one, in any of the four categories of biomarkers, stratified by the risk of bias; Note: Inflammatory biomarkers were separately examined within the same article. Abbreviations: Max, Maximum; Min, Minimum; Th2, T Helper 2

Seventy-one eligible studies evaluated the association between vitamin D status and inflammatory biomarkers based on measurements taken at a single point in time at the start of the study. These included 3 interventional studies: 2 RCTs [38, 45] and 1 crossover trial [56], as well as 68 observational studies, namely: 8 cohort studies [29, 30, 44, 46, 57–60], 10 case-control studies [31, 39, 40, 42, 47, 61–65], and 50 cross-sectional studies [32–37, 41, 43, 48–50, 66–104] (Table 1). Turkey contributed 11 studies (15.5%), followed by Egypt (8 studies, 11.3%) and Iran (5 studies, 7.0%). Other notable contributing countries were China, Poland, and United States (4 studies each, 5.6%). Most studies were published in the past 6 years (Fig. 2A). They were conducted predominantly in children (*N* = 50), with the remaining focusing on adults (*N* = 18) or both pediatric and adult patients (*N* = 3), with ages ranging from 0 to 80 years (Fig. 2B). The sample sizes of included studies varied widely, ranging from 13 to 847 individuals; collectively, these studies involved 7,787 participants (Fig. 2C). Vitamin D status (25(OH)D) was measured in all included studies, with a reported mean or median baseline values generally above 25 nmol/L, ranging from 29.5 nmol/L [92] to 131.0 nmol/L [80] with the exception of two studies in which lower values were observed: one study with a median (interquartile range) of 18.5 nmol/L (10.0, 26.0) in a group of individuals with uncontrolled asthma [66], and another study showing a mean ± standard deviation of 20.6 ± 2.0 nmol/L in a group of children [79]. In the remaining 16 studies [30, 32, 35, 39, 41, 43, 50, 64, 73, 88–90, 97, 102–104], the mean or median baseline 25(OH)D was not reported. As for vitamin D status categories, most studies (*N* = 34) adopted the following classification: deficiency (< or ≤ 50 nmol/L), insufficiency (50–75 nmol/L), and sufficiency (> or ≥ 75 nmol/L). Two studies used an alternative classification namely, deficiency (< or ≤ 25 nmol/L), insufficiency (25–50 nmol/L), and sufficiency (> or ≥ 50 nmol/L) [60, 96]. Ten studies adopted a dichotomic classification; 6 studies defining deficiency (< 50 nmol/L) and sufficiency (> or ≥ 50 nmol/L) [38, 45, 84, 86–88], whereas four studies defined deficiency or insufficiency as values < 75 nmol/L and sufficiency as values > or ≥ 75 nmol/L [34, 48, 49, 63]. Finally, thirteen studies adopted various other classifications, while eight studies not using any specific classification criteria [33, 41, 57, 70, 85, 93, 102, 103].

### Statistical analysis and adjustment variables

Of 71 studies, only 14 (19.7%) conducted a multivariate analysis for at least one inflammatory biomarker, adjusting for potential confounders such as age, sex, body mass index, ethnicity, inhaled corticosteroids, season/month of blood collection, sun exposure, vitamin D supplementation, vitamin D binding protein level, socioeconomic status, smoking status, alcohol consumption, upper respiratory tract infections, pulmonary function, and/or asthma status [29–32, 34, 46, 57, 66, 73, 83, 86, 89, 95, 103]. Other univariate statistical analyses were also utilized, mainly the Pearson and Spearman correlation coefficients (r/ρ) (45 studies), univariate linear regression (4 studies), Student’s t test (6 studies), Mann‒Whitney U test (6 studies), analysis of variance (ANOVA) (10 studies), Kruskal‒Wallis test (5 studies) and chi-square or Fisher test (4 studies) (Fig. 2D).

### Study quality assessment

Eight (3 cohort, 1 case‒control, and 4 cross-sectional) studies (11.3%) were rated at low risk of bias. These studies’ methodology was strengthened by their use of multiple regression models to adjust for confounding variables. Conversely, 13 (1 case‒control and 12 cross-sectional) studies (18.3%) were rated as having a high risk of bias; and the remining 50 (70.4%), at a moderate (neutral) risk of bias. Factors limiting the methodological quality of these studies included insufficient reporting of study inclusion & exclusion criteria and/or of data collection methods, lack of adjustment for confounders, no/limited discussion about biases and limitations, and unclear declarations of conflicts of interest. The methodological quality of included studies is detailed in Additional file 2: Table S1.

### Th2 pro-inflammatory biomarkers

**Fig. 3:**
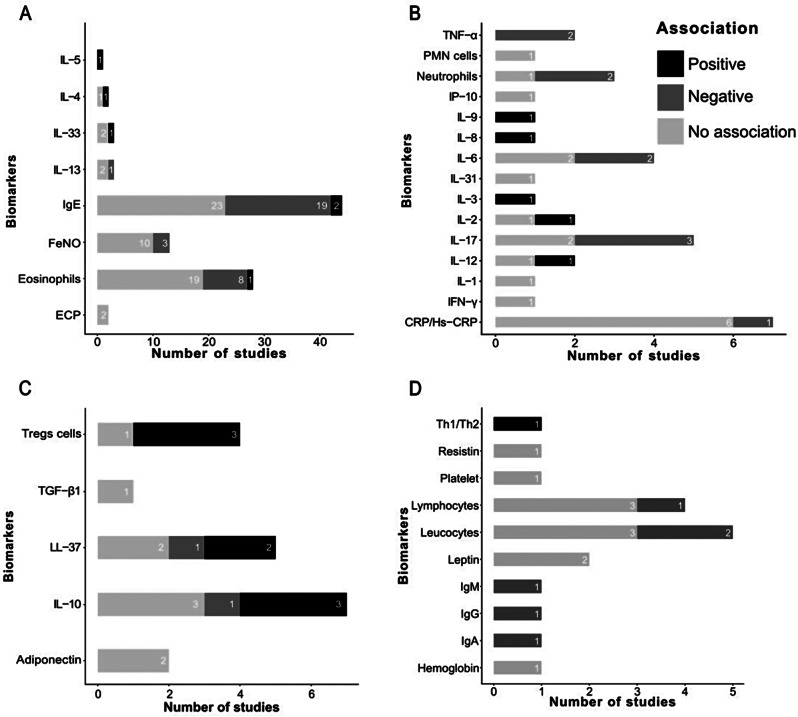
Summary of the type of association for inflammatory biomarkers explored in 71 included studies. (**A**) Th2 pro-inflammatory biomarkers (*N* = 58 studies). (**B**) Non-Th2 pro-inflammatory biomarkers (*N* = 18 studies). (**C**) Anti-inflammatory biomarkers (*N* = 16 studies). (**D**) Non-specific biomarkers (*N* = 10 studies). Abbreviations: CRP, C-reactive protein; ECP, Eosinophil Cationic Protein; FeNO, Fractional exhaled nitric oxide; Hs-CRP, High-sensitivity C-reactive protein; IFN-γ, Interferon gamma; Ig, Immunoglobulin; IL, Interleukin; IP-10, Interferon gamma-induced protein 10; LL-37, Cathelicidin; N, Number of total studies; PMN, Polymorphonuclear; TGF-β1, Transforming growth factor-beta 1; Th, T helper ; TNF-α, Tumor necrosis factor α; Tregs, Regulatory T cells

Most (58 of 71 (81.7%)) studies assessed the association between vitamin D status and at least one Th2 pro-inflammatory biomarker (Fig. 2E). We distinguished 44 studies on serum IgE, 28 on eosinophils (26 on blood and 2 on sputum), 13 on FeNO, three each on IL-13 (two in serum, one in bronchoalveolar lavage) and IL-33 (one in serum, one in plasma, and one in nasopharyngeal fluid), two each on serum IL-4 and ECP, and one on serum IL-5. These included 1 RCT, 5 cohort studies, 6 case‒control studies, and 46 cross-sectional studies [29–37, 41–49, 59, 61, 62, 64, 67–79, 81–102, 104]. Regardless of the risk of bias, the interventional study (1 RCT) did not detect a significant association between 25(OH)D and Th2 pro-inflammatory biomarkers. In observational studies, most comparisons (*N* = 58) also failed to detect significant associations. However, among those with statistically significant results, most indicated a negative association between 25(OH)D and Th2 pro-inflammatory biomarkers (*N* = 31), while a few (*N* = 6) revealed a positive association (Fig. 3A; Table 1). Only 7 (12.1%) of the 58 studies on Th2 pro-inflammatory biomarkers were rated as having a low risk of bias and focused on serum IgE, blood and sputum eosinophils, FeNO, and interleukins [29, 31, 32, 46, 73, 86, 95] (Fig. 2F).

#### Serum IgE

**Fig. 4:**
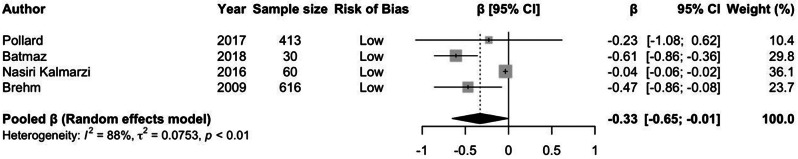
Forest plots for the association between vitamin D status (25(OH)D) (ng/mL) and serum IgE (IU/mL). Each study β is presented as a small vertical line with 95% CIs (horizontal lines). The weight of each study in the overall estimate is depicted graphically as the shaded box around each β estimate and numerically in the right-hand column. The pooled β and 95% CI, calculated with the random-effects model, are depicted by a lozenge. Heterogeneity was quantified by I^2^. Abbreviations: β: Linear coefficient of regression; CI: confidence intervals; IgE: immunoglobulin E; 25(OH)D, 25-Hydroxyvitamin D

**Fig. 5:**
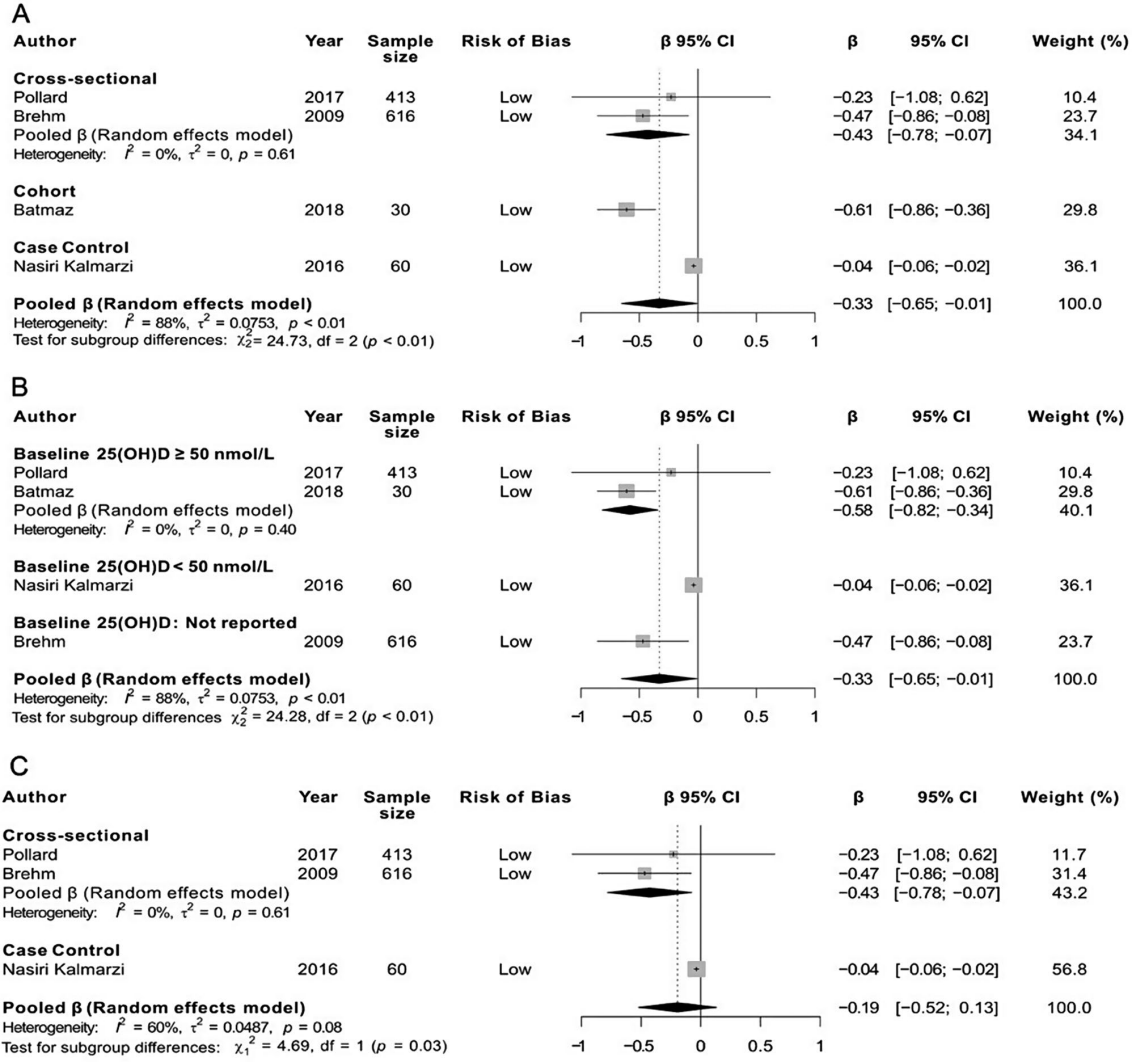
Forest plots for the association between vitamin D status (25(OH)D) (ng/mL) and serum IgE (IU/mL). (**A**) Adjusted β by study design. (**B**) Adjusted β by baseline 25(OH)D. (**C**) Adjusted β in sensitivity analysis after excluding studies with an asymmetric distribution of data. Each study β is presented as a small vertical line with 95% CIs (horizontal lines). The weight of each study in the overall estimate is depicted graphically as the shaded box around each β estimate and numerically in the right-hand column. The pooled β and 95% CI, calculated with the random-effects model, are depicted by a lozenge. Heterogeneity was quantified by I^2^. Abbreviations: β: Linear coefficient of regression; CI: confidence intervals; IgE: immunoglobulin E; 25(OH)D, 25-Hydroxyvitamin D

**Fig. 6:**
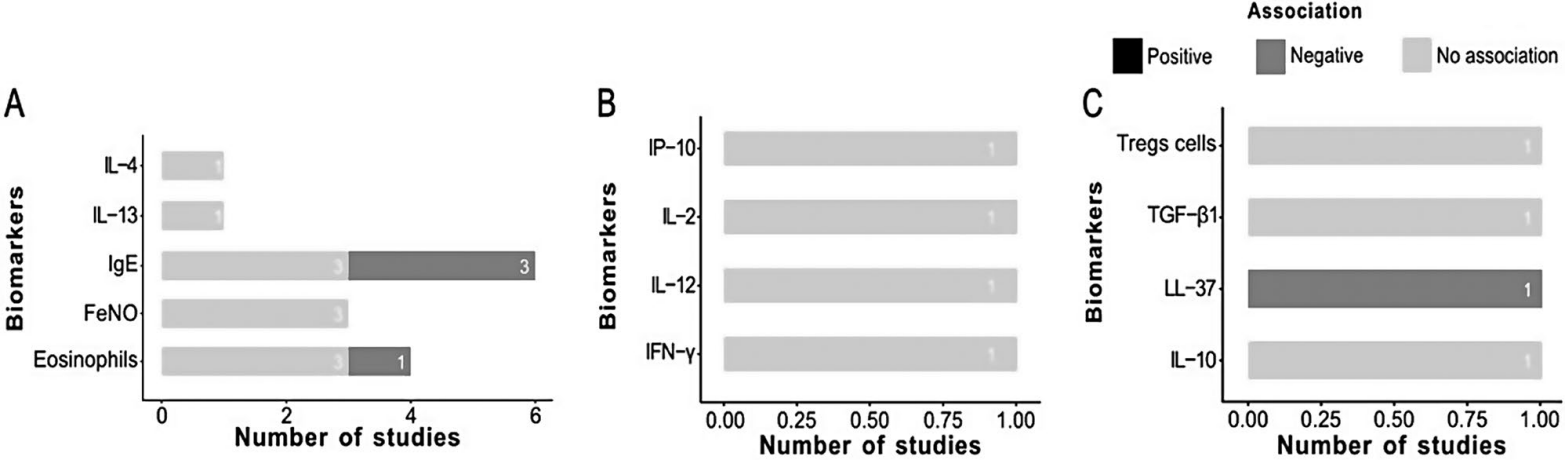
Summary of the type of association for inflammatory biomarkers explored in low-risk bias studies. (**A**) Pro-inflammatory biomarkers (Th2 inflammation) (*N* = 7). (**B**) Pro-inflammatory biomarkers (non-Th2 inflammation) (*N* = 2). (**C**) Anti-inflammatory biomarkers (*N* = 2). Abbreviations: FeNO, Fractional exhaled nitric oxide; IFN-γ, Interferon gamma; Ig, Immunoglobulin; IL, Interleukin; IP-10, Interferon gamma-induced protein 10; LL-37, Cathelicidin; N, Number of total studies; TGF-β1, Transforming growth factor-beta 1; Tregs, Regulatory T cells

Six (13.6%) studies on serum IgE were rated at low risk of bias [29, 31, 32, 46, 73, 95]; all were conducted in children. Study sample sizes varied from 30 [29] to 616 [32] participants; their mean baseline 25(OH)D levels ranged from 44.9 nmol/L [31] to 64.2 nmol/L [29]. Due to discrepancies in the parameterization of vitamin D and inflammatory biomarkers (i.e., continuous vs. categorical variables with different cut-off values), and the lack of reporting values on association estimates or 95% confidence intervals (CIs) for certain biomarkers [46, 73], our quantitative meta-analysis aggregated four of six low-risk studies [29, 31, 32, 95]. Using the random-effects model, the pooled estimate showed a significant negative association (β (95% CI) = − 0.33 (–0.65 to − 0.01); I^2^ = 88%; *P* < 0.01; *N* = 4 studies) with significant heterogeneity (Fig. 4). The association was affected by: (i) study design, with a stronger correlation between 25(OH)D and serum IgE in the cohort study and cross-sectional studies, than in the case-control study (Fig. 5A) and (ii) baseline vitamin D levels, with a stronger association in studies with mean baseline 25(OH)D ≥ 50 nmol/L or unknown, than in those with vitamin D insufficiency (Fig. 5B). Subgroup analyses based on patient age could not be conducted, as all included studies pertained to children. A sensitivity analysis that included the three studies with symmetrically distributed data confirmed the inverse association between 25(OH)D and IgE but failed to reach statistical significance (β (95% CI) = − 0.19 (–0.52 to 0.13); I^2^ = 60%; *P* = 0.08; *N* = 3 studies) (Fig. 5C). Of note, two small low-risk studies (one cohort and one cross-sectional) that reported their results narratively could not be aggregated in the meta-analysis [46, 73]; they mentioned no statistically significant association between 25(OH)D levels and serum IgE levels. In summary, focusing only on studies at low risk of bias on serum IgE, three reported a statistically significant negative association, while one large study and two small studies (that did not provide numerical values) concluded to no statistically significant association between 25(OH)D and serum IgE levels (Fig. 6A).

**Table 1:** Characteristics and results of studies on vitamin D status and inflammatory biomarkers in asthma patients

Reference	Country	Individuals, *n* (age, % male)	Vitamin D status (25(OH)D, % deficiency) (nmol/L) †	Inflammatory biomarkers ‡	Statistical analysis	Risk of bias
**Interventional studies (RCTs)** (***N*** = **3**)						
Kerley et al. [45], 2016	Ireland	44 (6–16 years old, male = 52.3%)	51.0 (24.0–80.0), **D (< 50) =** 50.0%	→ Serum IgE, Hs-CRP and ECP	Univariate	Moderate
Shabana et al. [38], 2019	Egypt	79 (> 19, 34.7 ± 7.2 years old, male = 64.5%)	44.6 ± 7.0, **D (< 50) =** NR	↓ Serum IL-17 A, → Serum IL-10	Univariate	Moderate
Thijs et al. [56], 2015	Germany	19 (18–45 years old, male = 26.3%)	65.0 ± 31.0, **D (< 30) =** 5.3%	→ Nasal secretion of LL-37	Univariate	Moderate
**Cohort studies** (***N*** = **8**)						
Arikoglu et al. [46], 2015	Turkey	67 (7–17 years old, male = 64.2%)	53.1 ± 26.1, **D (< 50) =** 59.7%	↓ Serum LL-37, → Serum IgE and blood eosinophils	Univariate & Multivariate	**Low**
Arikoglu et al. [57], 2017	Turkey	63 (10.7 ± 3.1 years old, male = 65.1%)	32.4 and 61.1 (12.5–124.8), **D** = NR	→ Serum IP-10	Univariate & Multivariate	**Low**
Bantulà et al. [58], 2022	Spain	37 (53 (43, 60) years old (non-obese), 56 (51, 59) years old (obese), male = 21.6%)	50.0 and 50.2 (35.2–79.7), **D (< 30) =** NR	→ Serum adiponectin and leptin	Univariate	Moderate
Batmaz et al. [29], 2018	Turkey	30 (7–17 years old, male = 66.6%)	64.2 ± 21.2, **D (≤ 50) =** NR	↓ Serum IgE, ↑ Treg cells, → Blood eosinophils, Serum IL-2, IL-4, IL-10, IL-12, IL-13, TGF-β1, and IFN-γ	Univariate & Multivariate	**Low**
Bose et al. [59], 2013	USA	121 (2–6 years old, male = 60.0%)	70.0 (52.4, 92.4), **D (≤ 50) =** 23.1%	↓ Serum IgE	Univariate	Moderate
Goleva et al. [30], 2012	USA	103 (32.5 (22.0, 46.0) years old (adults), 10 (8.5, 13.0) years old (children), male = 40.8%)	NR, **D (< 50) =** 47.6%	↓ Serum IgE, ↑ Serum LL-37	Multivariate	Moderate
Haag et al. [44], 2018	Germany	24 (4.92 ± 0.15 years old, male = 62.5%)	47.1 ± 5.3, **D (< 50) =** 20.8%	↑ Nasopharyngeal fluid of IL-33	Univariate	Moderate
Hebbar et al. [60], 2014	USA	13 (0–18 years old, male = NR)	42.2 (24.0, 44.2), **D (< 25) =** 30.8%	→ Plasma LL-37	Univariate	Moderate
**Case Control studies** (***N*** = **10**)						
Ahmed et al. [61], 2020	Egypt	50 (12.7 ± 3.3 years old, male = 56.0%)	33.6 ± 26.2, **D (≤ 50) =** 72.0%	↓ Serum IgE	Univariate	Moderate
Al-Athari et al. [62], 2022	Iraq	127 (19–59 years old, male = 45.1%)	51.0 ± 17.5, **D (< 50) =** 7.8%	→ Serum Hs-CRP and FeNO	Univariate	Moderate
Albanna et al. [63], 2012	Egypt	30 (3–13 years old, male = 30.0%)	63.0 ± 24.5, (**< 75**) = 70.0%	↑ Plasma LL-37, → Serum Hs-CRP	Univariate	High
Ehlayel et al. [42], 2011	Qatar	483 (7.0 ± 3.8 years old, male = 51.1%)	42.9 ± 27.5, **D (< 50) =** 25.0%	↑ Serum IgE	Univariate	Moderate
Kilic et al. [47], 2019	Turkey	100 (5–18 years old, male = 52.0%)	55.4 ± 30.7, **D (< 50) =** 58.4%	→ Serum IgE, blood eosinophils, and FeNO	Univariate	Moderate
Maalmi et al. [40], 2012	Tunisia	39 (6–16 years old, male = 68.4%)	52.1 ± 18.7, **D (< 50) =** 43.6%	↓ Plasma IL-17, ↑ Th1/Th2 ratio, Treg cells, and plasma IL-10, → Plasma IL-6	Univariate	Moderate
Mohammadzadeh et al. [64], 2020	Iran	100 (2–13 years old, male = 50.0%)	NR, **D (< 50) =** 45.0%	↓ Serum IgE, → Blood eosinophils and WBC	Univariate	Moderate
Nasiri Kalmarzi et al. [31], 2016	Iran	60 (6–18 years old, male = 58.3%)	44.9 ± 21.7, **D (< 50)** = 43.3%	↓ Serum IgE	Univariate & Multivariate	**Low**
Pervaiz et al. [39], 2019	Turkey	30 (5–15 years old, male = 70.0%)	NR, **D (≤ 50) =** 56.6%	↓ Serum IL-17, → Serum IL-6	Univariate	Moderate
Wawrzyniak et al. [65], 2017	Poland	25 (8 (6–12) years old, male = 68.0%)	56.9 ± 24.0, **D (≤ 50) =** 57.0%	→ Lymphocytes (CD3, CD19, CD4, CD8, CD4/CD8, CD16/CD56, NKT)	Univariate	Moderate
**Cross sectional studies** (***N*** = **50**)						
Adam-Bonci et al. [48], 2020	Romania	131 (0–18 years old, male = 54.2%)	61.7 (26.5) (≥ 5 years), 83.2 (45.5) (< 5 years), **< 75 =** 58.8%	→ Serum IgE and blood eosinophils	Univariate	Moderate
Al-Attas et al. [50], 2017	Saudi Arabia	58 (14.2 ± 0.3 years old, male = 63.8%)	NR, **D (< 50) =** 44.0%	→ Serum adiponectin, resistin and leptin	Univariate	Moderate
Aldubi et al. [66], 2015	Saudi Arabia	45 (4–18 years old, male = 60.0%)	52.7 (47.8, 67.2) (controlled asthma), 33.3 (27.0, 35.2) (partial controlled asthma), 18.5 (10.0, 26.0) (uncontrolled asthma), **D (< 50) =** 77.8%	↑ Serum IL-10	Multivariate	Moderate
Al-Thagfan et al. [33], 2021	Saudi Arabia	73 (44.2 ± 13.6 years old, male = 78.1%)	36.7 ± 8.6, **D** = NR	↓ Serum IgE and **b**lood eosinophils	Univariate	Moderate
Alyasin et al. [67], 2011	Iran	50 (6–18 years old, male = 62.0%)	123.0 ± 53.5, **D (< 50) =** 4.0%	→ Blood eosinophils	Univariate	Moderate
Alzughaibi et al. [68], 2022	Iraq	127 (33.4 ± 13.0 years old, male = 46.6%)	50.9 ± 17.5, **D (< 30) =** 7.8%	→ FeNO	Univariate	Moderate
Amorim et al. [69], 2020	Brazil	26 (6–12 years old, male = 73.1%)	60.0 (47.4, 77.4), **< 60.0 =** 50.0%	↓ Blood eosinophils and serum IgE	Univariate	Moderate
Beyhan-Sagmen et al. [43], 2017	Turkey	106 (35.5 ± 9.4 years old, male = 36.8%)	NR, **D (< 25) =** 66.0%	↑ Serum IgE, → Blood eosinophils	Univariate	Moderate
Brehm et al. [25], 2009	Costa Rica	616 (8.7 (7.6, 10.5) years old, male = 60.0%)	NR (31.2–245.2), **D (< 50) =** 3.4%	↓ Blood eosinophils and serum IgE	Univariate & Multivariate	**Low**
Brehm et al. [49], 2012	Puerto Rico	287 (6–14 years old, male = 60.0%)	79.9 ± 20.0, **< 75 =** 44.0%	→ Serum IgE	Univariate	Moderate
Beigh et al. [70], 2020	India	150 (31.0 ± NR years old, male = 35.3%)	47.0 ± 23.3, NR	↓ Serum IgE	Univariate	Moderate
Bonanno et al. [71], 2014	Italy	35 with rhinitis (8–13 years old, male = 62.8%)	58.6 (52.4–67.6), **D (< 50) =** 17.1%	↓ Blood eosinophils and serum IgE, → Plasma IL-33 and IL-31	Univariate	High
Chary et al. [72], 2016	India	60 (2–6 years old, male = 65.0%)	38.8 ± 1.7, **D (< 37.5) =** 80.0%	↑ Treg cells, ↓ B lymphocyte (CD23-CD21), → Serum IgE	Univariate	High
Checkley et al. [73], 2015	Peru	86 (13–15 years old, male = NR)	NR	→ Serum IgE and FeNO	Multivariate	**Low**
Dabbah et al. [74], 2015	Israel	71 (6–18 years old, male = 65.0%)	57.5 ± 19.3, **D (< 50) =** 36.6%	→ Blood eosinophils, serum IgE, Hs-CRP and FeNO	Univariate	Moderate
Dajic et al. [75], 2019	Serbia	150 (0–18 years old, male = 57.3%)	51.1 ± 20.5, **D (< 50) =** NR	→ Serum IgE	Univariate	High
Dogru et al. [76], 2014	Turkey	120 (4.4 ± 1.2 years old, male = 60.8%)	53.7 ± 19.3, **D (≤ 50) =** 43.3%	→ Blood eosinophils and serum IgE	Univariate	Moderate
Ebrahimi et al. [77], 2021	Iran	84 (9.0 ± 2.6 years old, male = 54.8%)	61.3 ± 29.0, **D (< 25) =** NR	↓ Blood eosinophils and serum IgE	Univariate	Moderate
Elnady et al. [78], 2013	Egypt	50 (4–15 years old, male = 72.0%)	62.7 ± 8.0, **D (< 62.5) =** 40.0%	↓ Blood eosinophils and serum IgE	Univariate	High
El-Said et al. [79], 2016	Egypt	60 (6–45 years old, male = NR)	52.6 ± 10.7 (adult) and 20.6 ± 2.0 (children), **D (≤ 50) =** 40.0%	↓ Serum IgE	Univariate	High
Gupta et al. [41], 2014	UK	35 (5–16 years old, male = 45.7%)	NR	↑ Airway lavage of IL-10, → Airway lavage of IL-13 and IL-17 A	Univariate	High
Hakamifard et al. [80], 2020	Iran	30 (5–12 years old, male = 46.7%)	131.0 ± 45.4, **D (< 25) =** NR	→ Blood Hb, WBC, Platelet, PMN	Univariate	High
Hamed et al. [81], 2016	Egypt	60 (6–16 years old, male = 45.0%)	109.7 ± 67.6, **D (< 50) =** 0.0%	↑ Blood eosinophils	Univariate	Moderate
Havan et al. [82], 2017	Turkey	72 (6–18 years old, male = 47.2%)	36.0 ± 15.5, **D (< 37.5) =** 52.8%	→ Blood eosinophils and serum IgE	Univariate	Moderate
Hutchinson et al. [83], 2016	Ireland	100 (53 ± 1.5 years old, male = 47.0%)	41.0 ± 25.0, **D (< 30) =** 45.0%	→ Serum Hs-CRP, IgE and ECP	Multivariate	Moderate
Hutchinson et al. [84], 2018	Ireland	44 (6–16 years old, male = 52.3%)	51.0 (24.0–80.0), **D (< 50) =** 50.0%	↓ Serum IgE	Univariate	Moderate
Janeva-Jovanovska et al. [85], 2017	Macedonia	30 (20–71 years old, male = 36.7%)	38.1 ± 14.5, NR	→ Serum IL-33	Univariate	Moderate
Jolliffe et al. [86], 2018	UK	297 (48.7 ± 14.4 years old, male = 42.8%)	50.6 ± 24.9, **D (< 25) =** 13.5%	→ Sputum eosinophils and FeNO	Univariate & Multivariate	**Low**
Kalicki et al. [87], 2017	Poland	34 (8 (6, 12) years old, male = NR)	57.4 ± 24.0, **D (< 50) =** 56.0%	→ Serum IgE, Treg cells and FeNO	Univariate	High
Korn et al. [34], 2013	Germany	280 (45.0 ± 13.8 years old, male = 40.0%)	63.9 ± 29.4, **I (< 75) =** 67.0%	↓ Sputum eosinophils and FeNO, → Serum IgE and IL-10,	Univariate & Multivariate	Moderate
Kuti et al. [36], 2021	Nigeria	90 (2–15 years old, male = 62.2%)	85.0 ± 35.7, **D (< 50) =** 16.7%	↓ Serum IL-6, IL-10, IL-13, → Serum IL-1β and IL-17, ↑ Serum IL-2, IL-3, IL-4, IL-5, IL-8, IL-9, and IL-12	Univariate	Moderate
Lan et al. [88], 2014	China	32 (47.5 ± 12.7 years old, male = 46.9%)	NR, **D (≤ 50) =** 50.0%	↓ Serum TNF-α, → Blood eosinophils, leucocytes, neutrophils, and lymphocytes	Univariate	High
Li et al. [89], 2011	China	435 (1–18 years old, male = 38.4%)	NR (9–85), **D (< 50) =** 89.0%	→ Blood eosinophils and serum IgE	Univariate & Multivariate	Moderate
Montero-Arias et al. [90], 2013	Costa Rica	121 (48.1 ± 15.7 years old, male = 16.5%)	NR, **D (< 50) =** 29.7%	→ Blood eosinophils and serum IgE	Univariate	Moderate
Osman et al. [91], 2019	Egypt	100 (2–5 years old, male = 68.0%)	82.3 ± 56.9, **D (< 50) =** 32.0%	↓ Leucocytes, → Blood eosinophils and serum IgE	Univariate	Moderate
Ozdogan et al. [92], 2017	Turkey	71 (7–17 years old, male = 50.7%)	29.5 ± 25.7, NR	↓ Serum IgE	Univariate	Moderate
Ozkars et al. [93], 2019	Turkey	59 (7–14 years old, male = 55.9%)	41.7 ± 12.9, NR	→ FeNO	Univariate	High
Ozturk Thomas et al. [94], 2019	Turkey	73 (6–15 years old, male = 54.8%)	30.2 ± 18.5, **D (< 50) =** 82.2%	→ Serum IgE	Univariate	Moderate
Pollard et al. [95], 2017	Peru	413 (9–19 years old, male = 56.7%)	62.9 ± 25.2, **D (< 50) =** NR	→ Serum IgE and FeNO	Multivariate	**Low**
Protsiuk et al. [96], 2018	Ukraine	106 (15.8 ± 1.8 years old, male = NR)	40.6 ± 13.6, **D (≤ 25) =** 32.0%	→ Serum IgE	Univariate	High
Samaha et al. [35], 2015	Egypt	40 (38.8 ± 10.0 years old, male = 37.5%)	NR, **D (≤ 50) =** 22.0%	↓ Serum IgE and FeNO	Univariate	Moderate
Santos et al. [97], 2018	Brazil	60 (7–14 years old, male = 57.0%)	NR, **D (< 37.5) =** NR	→ Serum IgE	Univariate	Moderate
Searing et al. [98], 2010	USA	100 (0–18 years old, male = 64.0%)	77.4 ± NR, **D (< 50) =** 17.0%	↓ Serum IgE, → Blood eosinophils	Univariate	Moderate
Solidoro et al. [99] 2017	Italy	119 (52 (13–80) years old, 21.0%)	41.3 ± 1.7, **D (< 50) =** 76.0%	→ Blood eosinophils and FeNO	Univariate	Moderate
Odrowąż-Sypniewska et al. [100], 2014	Poland	116 (2–12 years, male = 53.4%)	69.7 ± 21.0, **D (≤ 50) =** 19.1%	↓ Blood neutrophils, → Serum IgE, Hs-CRP, and blood eosinophils	Univariate	Moderate
Tamašauskienė et al. [101], 2015	Lithuania	85 (46.4 ± 1.5 years old, male = 30.5%)	35.8 ± 1.4, **D (< 25) =** NR	→ Blood eosinophils and serum IgE	Univariate	High
Wang et al. [37], 2018	China	60 (4.3 ± 1.4 years old, male = 48.3%)	35.7 ± NR, **D (< 35.7) =** 53.3%	↓ Blood eosinophils, neutrophils, leucocytes, and serum TNF-α, IL-6, IgG, IgM, and IgA, → Lymphocytes	Univariate	Moderate
Wytrychowski et al. [102], 2020	Poland	63 (49.9 ± 14.3 years old, male = 44.5%)	NR	↓ FeNO	Univariate	Moderate
Yang et al. [103], 2020	China	847 (49.0 ± 19.0 years old, male = 36.5%)	NR	↓ Serum CRP	Multivariate	Moderate
Yousif et al. [104], 2019	Iraq	61 (6–18 years old, male = 57.4%)	NR, **D (< 50) =** 59.0%	↓ Serum IgE	Univariate	Moderate

↓: Negative association; → : No association; ↑ : Positive association

Abbreviations: D, Deficiency; n, number; NR, Not Reported; RCT, Randomized Controlled Trial; UK, United Kingdom; USA, United States of America

†Values are reported as number (percentage %), mean ± standard deviation, median (first interquartile, third interquartile), median (IQR), median (minimum-maximum)

‡CD, Cluster of Differentiation; CRP, C-reactive protein; ECP, Eosinophil cationic protein; FeNO, Fractional exhaled Nitric Oxide; Hb, Hemoglobin; Hs-CRP, High-sensitivity C-reactive protein; IFN-γ, Interferon Gamma; Ig, Immunoglobulin; IL, Interleukin; IP-10, IFN-gamma-inducible protein 10; LL-37, Cathelicidin; NKT, Natural Killer T cells; PMN, Polymorphonuclear cells; TGF-β1, Transforming Growth Factor Beta 1; Th, T helper cells; TNF-α, Tumor Necrosis Factor Alpha; Treg, Regulatory T cells; WBC, White Blood cells

#### Blood and sputum eosinophils

Only three (11.5%) studies, all reporting blood eosinophils, were rated at low risk of bias [29, 32, 46] but aggregation of these studies was not feasible. A large cross-sectional study demonstrated a statistically significant negative association in 616 children whose baseline 25(OH)D levels ranged from 31.2 to 245.2 nmol/L (β (95% CI) = − 0.26 (− 0.48 to − 0.05)) [32]. One small cohort study reported no statistically significant association between 25(OH)D levels and blood eosinophils in 67 children, with a mean baseline 25(OH)D level of 53.1 nmol/L [46]. Another small cohort study found no statistically significant association in 30 children with a mean baseline 25(OH)D level of 64.2 nmol/L (*r* = 0.009; p value = 0.923) [29]. Regarding sputum eosinophils, only one cross-sectional study at low risk of bias was identified, involving 297 adults; no significant group difference in sputum eosinophil (%) (β (95% CI) = 1.03 (0.58 to 1.84)) was observed between individuals with 25(OH)D levels < 50 nmol/L vs. ≥50 nmol/L [86]. In summary, one large study at low risk of bias reported a negative association between 25(OH)D and serum eosinophils, whereas two small studies failed to identify a significant association; no statistically significant association with sputum eosinophils was noted in a large adult study.

#### FeNO

We identified three (23.1%) cross-sectional studies at low risk of bias that evaluated the association between vitamin D status and FeNO. Two of them reported mean 25(OH)D levels greater than 50 nmol/L [86, 95], while other failed to comment on baseline values [73]. No meta-analysis was feasible. One pediatric study of 86 children narratively mentioned the absence of a statistically significant association [73]. Another study involving 413 children, presented no statistically significant association (β (95% CI = − 0.007 (− 0.036 to 0.002)) [95]. The third study in 297 adults comparing FeNO across various serum 25(OH)D levels found no statistically significant differences (< 25 nmol/L (β (95% CI) = 1.04 (0.80 to 1.35)); 25-49.9 nmol/L (β (95% CI) = 1.10 (0.90 to 1.34)); 50-74.9 nmol/L (β (95% CI) = 1.13 (0.91 to 1.40)), compared to 25(OH)D ≥ 75 nmol/L serving as the reference category [86]. In conclusion, no statistically significant associations between FeNO and 25(OH)D were reported in two adult and one pediatric studies at low risk of bias.

#### Interleukins

We identified one small cohort study at low risk of bias investigating the association between 25(OH)D and two serum interleukins in 30 children with a baseline 25(OH)D level of 64.2 nmol/L [29]; no statistically significant association between 25(OH)D levels and serum IL-4 (β (95% CI) = − 0.10 (–0.80 to 0.46)) or serum IL-13 (*r* = 0.128; *p* = 0.099) was observed [29].

### Non-Th2 pro-inflammatory biomarkers

Eighteen (25.4%) of 71 studies explored the association between pro-inflammatory biomarkers of non-Th2 inflammation and vitamin D, namely 2 RCTs, 2 cohort studies, 4 case‒control studies, and 10 cross-sectional studies [29, 36–41, 45, 57, 62, 63, 71, 74, 80, 83, 88, 100, 103]. We identified seven studies testing serum Hs-CRP/CRP, five exploring IL-17 in the serum (*N* = 3), plasma (*N* = 1), and airway lavage (*N* = 1), four tested serum (*N* = 3) and plasma (*N* = 1) IL-6, three on blood neutrophils, two each on serum TNF-α, IL-2, and IL-12, and one each on serum IL-3, IL-8, IL-9, IL-1, IFN-γ, IP-10, PMN cells and plasma IL-31. Irrespective of the risk of bias, in interventional studies, 1 RCT did not detect a significant association between 25(OH)D and non-Th2 pro-inflammatory biomarkers, while another detected a negative association. In observational studies, the majority (*N* = 17) of comparisons did not find any statistically significant association. However, some comparisons (*N* = 9) showed a negative association between 25(OH)D and non-Th2 pro-inflammatory biomarkers, while the remainder (*N* = 5) indicated a positive association (Fig. 3B; Table 1). Only two (11.1%) of these 18 studies on non-Th2 pro-inflammatory biomarkers were rated as having a low risk of bias [29, 57].

These studies evaluated the association between 25(OH)D and serum IP-10, IL-2, IL-12, and/or IFN-γ levels [29, 57]. One cohort study was conducted in 63 children with a median baseline 25(OH)D levels of 32.4 nmol/L in the acute asthma group and 61.1 nmol/L in the controlled asthma group; it demonstrated no significant association with serum IP-10 levels (β (95% CI): 1.70 (− 2.6 to 6.0)) [57]. Another cohort study involving 30 children with a baseline 25(OH)D level of 64.2 nmol/L also showed no statistically significant associations between 25(OH)D and serum IL-2 (*r* = − 0.226; *p* = 0.072), serum IL-12 (*r* = 0.151; *p* = 0.099), or serum IFN-γ (*r* = − 0.195; *p* = 0.064) levels [29]. Overall, in the two small studies at low risk of bias, no meta-analysis was feasible, and no statistically significant associations were observed (Fig. 6B).

### Anti-inflammatory biomarkers

Sixteen studies investigated the association between vitamin D status and anti-inflammatory biomarkers. These included 2 RCTs, 6 cohort studies, 2 case‒control studies, and 6 cross-sectional studies [29, 30, 34, 36, 38, 40, 41, 46, 50, 56, 58, 60, 63, 66, 72, 87]. Overall, seven studies examined the association between 25(OH)D and IL-10 in the serum (*N* = 5), plasma (*N* = 1) and bronchial lavage (*N* = 1). Five studies examined LL-37 measured in serum or plasma (two each) and one in nasal secretions; four focused on blood Treg cells, and one on serum TGF-β. When considering all studies irrespective of the risk of bias, and for interventional studies (2 RCTs), no statistically significant associations between serum 25(OH)D and anti-inflammatory biomarkers were detected. For observational studies, most comparisons (*N* = 8) reported a statistically significant positive association between serum 25(OH)D and anti-inflammatory biomarkers. This was closely followed by no significant association in 7 comparisons, and a negative association in the remaining 2 comparisons (Fig. 3C; Table 1). Only two studies on anti-inflammatory biomarkers were rated as having a low risk of bias [29, 46].

These studies evaluated the association between 25(OH)D and serum LL-37, blood Treg cells, and/or serum TGF-β. Both cohort studies were conducted in children with mean baseline 25(OH)D levels > 50 nmol/L [29, 46]. One small study (*N* = 67) demonstrated a statistically significant negative association between 25(OH)D levels and serum LL-37 (β (95% CI) = − 0.018 (–0.032 to − 0.003)) [46]. The other study found no statistically significant association between 25(OH)D levels and Treg cells (β (95% CI) = 0.41 (0.23 to 0.50), IL-10 (*r* = − 0.099; *p* = 0.283), or TGF-β (*r* = − 0.170; *p* = 0.064) [29] (Fig. 6C).

### Non-specific biomarkers

Ten of 71 (14.1%) studies evaluated the association between vitamin D status and non-specific biomarkers that are neither pro- nor anti-inflammatory (1 cohort study, 3 case‒control studies, and 6 cross-sectional studies) [37, 40, 50, 58, 64, 65, 72, 80, 88, 91]. No study was rated at being at a low risk of bias. Overall, the majority (*N* = 11) of investigations reported no statistically significant association with non-specific biomarkers; a minority of comparisons (*N* = 6) observed a statistically significant negative association, and one, a positive association with these biomarkers (Fig. 3D; Table 1).

## Discussion

This review summarizes 71 studies exploring the association between serum vitamin D status (25(OH)D) and inflammatory biomarkers in patients with asthma. Of these, only eight studies (11.3%) were rated at a low risk of bias and served as the main basis for our conclusions. A meta-analysis of 4 studies was possible for a single biomarker—a Th2 pro-inflammatory type—showing a statistically significant negative association between serum 25(OH)D levels and serum IgE levels. In the small number of remaining studies at low risk of bias, the diversity of biomarkers studied, the variety of metrics used, and incomplete reporting prevented the quantitative aggregation of data. The reporting of the studies’ direction of associations within the context of a narrative review provided some information. However, the inability to take into consideration the precision of results—that is, to distinguish between evidence of no effect and no evidence of effect due to insufficient power in many studies—made it challenging to draw firm conclusions about the true association between 25(OH)D serum levels and most biomarkers.

For Th2 pro-inflammatory biomarkers, our meta-analysis of four studies at low risk of bias revealed that for every 2.5 nmol/L (1 ng/mL) increase in 25(OH)D levels, serum IgE decreased by about 0.33 IU/mL. As subgroup analyses highlighted a stronger association in children with 25(OH)D levels above 50 nmol/L or unknown than others, the linearity of the association remains to be established across the whole spectrum of 25(OH)D levels. The two remaining small studies at low risk of bias that narratively reported no significant association, failed to provide numerical values of the effect’s magnitude or precision, thus preventing any worthwhile contribution to the conclusions. We were unable to find previous reviews examining the association between 25(OH)D levels and IgE, confirming a gap in the literature that our review begins to address. However, our results align with other observational studies involving patients with inflammatory diseases that have reported a negative association between 25(OH)D and serum IgE, although without adjusting for potential confounders. Indeed, in children with gastrointestinal food allergies and atopic dermatitis, an inverse correlation between vitamin D and IgE levels was noted, with correlation coefficients ranging from − 0.4 to −0.5 [105, 106]. As for eosinophils, although two small studies that could not be aggregated failed to identify a significant association, possibly due to lack of power, we identified one large study that found a statistically significant negative association between blood eosinophils and serum 25(OH)D; whereas the magnitude of the effect corresponded to a weak association, the lower limit (more negative) of the confidence interval indicated a moderate effect size. This observed negative association is supported by the results of a study conducted in children with gastrointestinal food allergy, where children with low 25(OH)D (< 75 nmol/L) had persistent blood eosinophilia compared to those with higher 25(O)HD (≥ 75 nmol/L) (56% vs. 25%, *P* < 0.05) [105]. No significant associations with other Th2 pro-inflammatory biomarkers, including FeNO, were observed, and we could not find any previous reviews examining the association between 25(OH)D levels and those biomarkers. Collectively, our analysis suggests a negative association between serum 25(OH)D and serum IgE, and possibly with blood eosinophils; no apparent association with FeNO; and insufficient data to conclude on other Th2 biomarkers in asthma.

When examining evidence from systematic reviews of vitamin D supplementation trials on Th2 inflammatory biomarkers, the findings do not suggest a causal relationship for the negative association between 25(OH)D and Th2 inflammatory biomarkers, that is, a significant decrease in these biomarkers following supplementation. Our 2024 systematic review and meta-analysis of 13 intervention studies—testing vitamin D supplementation (cholecalciferol, calcidiol, and calcitriol) doses ranging from 800 to 400,000 IU over periods of 6 weeks to 12 months—revealed no significant group difference at study endpoint compared to placebo, in serum IgE levels (4 RCTs, Mean difference [MD] [95% CI]: 0.06 [-0.13, 0.26] IU/mL), blood eosinophils (3 RCTs, MD [95% CI]: − 0.02 [-0.11, 0.07] 10^3^/µL), FeNO (3 RCTs, MD [95% CI]: -4.10 [-10.95, 2.75] ppb), or other Th2 interleukins namely serum [107], sputum [108], exhaled breath condensate IL-4 [109], and sputum IL-13 [108], among 1459 adults and children with asthma [27]. Our later findings are concordant with previous systematic reviews and meta-analyses of RCTs that included the same or fewer trials testing the impact of supplementation [110–112]. Due to insufficient reporting, we were unable to aggregate data to examine whether, compared to placebo, vitamin D supplementation was associated with significant within-patient decrease from baseline in Th2 biomarkers. However, a significant decrease in serum IL-13 was observed in 86 adult patients supplemented with a daily dose of calcitriol for 6 months [113]. Indeed, included most supplementation trials did not adjust for baseline values of biomarkers and potential confounders, nor did they report the within-patient change in biomarkers between groups. Consequently, it remains unclear whether the observed inverse cross-sectional association between 25(OH)D and serum IgE (and potentially blood eosinophils) in this review reflects a direct effect of vitamin D deficiency, an indirect effect through other mediators, or a spurious association.

As for non-Th2 pro-inflammation, studies at low risk of bias identified in this review did not find a statistically significant association between 25(OH)D levels and a variety of biomarkers, namely IP-10, IL-2, IL-12, or IFN-γ. No study at low risk of bias was identified for non-specific biomarkers, making it impossible to generate hypotheses. Additionally, no prior systematic reviews have been found on the association between 25(OH)D and either non-Th2 pro-inflammation or non-specific biomarkers in other inflammatory conditions. In a supplementation trial, no significant difference was observed at the 12-month endpoint in sputum IL-2 and IFN-γ levels in 250 adults supplemented bi-monthly with 120,000 IU of cholecalciferol compared to a placebo [108]. The paucity of studies at low risk of bias, including supplemental trials, prevent any firm conclusion regarding a potential association between vitamin D level or supplementation and non-Th2 pro-inflammatory biomarkers.

Regarding anti-inflammatory biomarkers, the only one (small) study at low risk of bias reported a statistically significant negative association between 25(OH)D levels and serum LL-37 in children, suggesting less antimicrobial activity in those with higher 25(OH)D levels; no statistically significant associations were found for IL-10, Treg cells, or TGF-β. Of note, a supplemental trial alluded to the opposite conclusion. Indeed, in 86 adults with asthma, supplementation with calcitriol for 6 months resulted in a significantly greater increase in sputum LL-37 and serum IFN-γ compared to the placebo group [113], suggesting more antimicrobial activity with greater 25(OH)D levels. This is in concordance with a study in primary cultures of normal human and cystic fibrosis bronchial epithelial cells, which showed that vitamin D up-regulates cathelicidin expression and enhances antimicrobial activity in both settings [114]. Overall, the evidence from our studies at low risk of bias and supplementation trials suggests that 25(OH)D levels are possibly associated with LL-37, but the direction of effect needs to be clarified.

Our findings revealed a negative association with IgE, and possibly with blood eosinophils and LL-37, consistent with recent mechanistic reviews. These reviews indicate that vitamin D reduces levels of pro-inflammatory cytokines such as IL-1, IL-6, and TNF-α [115, 116]. Moreover, evidence suggests that vitamin D influences B lymphocytes by inhibiting their differentiation, limiting their proliferation, and decreasing immunoglobulin production such as IgE, while also boosting IL-10 production, thereby exerting an additional regulatory effect [115, 116]. It is important to note the dual role of LL-37, which can exhibit both pro- and anti-inflammatory effects, either directly stimulating inflammatory cells or modulating the cellular response to specific cytokines or signals, which potentially explain the divergent findings. Remarkably, it can rapidly transition from an anti-inflammatory to a pro-inflammatory state under certain conditions [117, 118]. Our findings underscore the multifaceted role of vitamin D in modulating immune responses, highlighting its potential as a crucial regulator of both innate and adaptive immunity. Yet, the paucity of high-quality in vivo data prevents any firm conclusion about this validity of these purported mechanisms of action in asthma patients.

## Strengths and limitations

Our systematic review has several strengths and limitations. First, our study was comprehensive, as evidenced by our broad literature search across several databases, the incorporation of various study designs, adult and pediatric populations, and a wide range of inflammatory biomarkers. Whereas the variability in the populations studied, in age, comorbidities, vitamin D status categories, and asthma control status contributed to significant heterogeneity, it also increased the generalisability of our findings. We adhered to well-defined inclusion and exclusion criteria, independent quality assessment using a validated tool, systematic and rigorous approach to data extraction and assessment of analytic methods and focused our conclusions on studies at low risk of bias. However, the large body of evidence is weakened by the small number of studies at low risk of bias (e.g., no, or inadequate adjustment for confounding, incomplete reporting of study selection criteria and/or of data collection methods, use of various metrics that prevented aggregation). Whereas this review included intervention supplementation trials, most of them reported the 25(OH)D and biomarkers at baseline and not over time. Consequently, the cross-sectional association between 25(OH)D levels and some inflammatory biomarkers observed in this review, do not imply causation as they could be due to type 1 error or reverse causality. Indeed, we cannot rule out the possibility that uncontrolled asthma might be independently associated with both a rise in Th2 biomarkers and reduced 25(OH)D, if the later acts as an acute phase response to inflammation [119] or if uncontrolled disease is associated with reduced sun exposure [120, 121] and thus vitamin D synthesis [122]. Consequently, monitoring these biomarkers in the context of interventional vitamin D supplementation trial are crucial for tracking temporal changes and establishing support for or against a cause-and-effect relationship. Also, complete reporting of studies with high methodological quality, featuring larger sample sizes, robust designs, precise measurements, and strategies to minimize bias and reduced imprecision (e.g., by treating 25(OH)D levels as a continuous variable) are essential to better address the questions. This approach would facilitate the pooling of data for future meta-analyses, thereby offering more comprehensive insight into the in vivo mechanism of action of vitamin D in asthma patients by clarifying the direction, magnitude, and precision of associations.

## Conclusion

Most of the published evidence regarding the association between 25(OH)D and inflammatory biomarkers is at significant (high or moderate) risk of bias, resulting in a paucity of methodologically strong studies on which to base firm conclusions. Studies at low-risk of bias suggest a negative, but not robust, association between vitamin D status and serum IgE levels, a probably negative association with blood eosinophils and serum LL-37; collectively alluding to an immunomodulatory effect of vitamin D. Future interventional and longitudinal studies, adjusting for confounders, should examine the association over time between serum 25(OH)D levels and inflammatory biomarkers, to clarify our observations. Understanding this relationship is crucial for developing targeted interventions and clinical guidelines aimed at optimizing vitamin D levels to manage inflammation in asthma patients effectively.

## Electronic supplementary material

Below is the link to the electronic supplementary material.

Supplementary Material 1

Supplementary Material 2

## Data Availability

No datasets were generated or analysed during the current study.

## Abbreviations

25(OH): 25-hydroxyvitamin D
EBM: Evidence-Based Medicine
ANOVA: Analysis of variance
CINAHL: Cumulative Index to Nursing and Allied Health Literature
CRP: C-reactive protein
ECP: Eosinophil Cationic Protein
FeNO: Fractional exhaled nitric oxide
Hs-CRP: High-sensitivity C-reactive protein
IFN-γ: Interferon gamma
Ig: Immunoglobulin
IL: Interleukin
IP-10: Interferon gamma-induced protein 10
IOM: Institute of Medicine
LL-37: Cathelicidin
MESH: Medical Subject Headings
OCS: Oral corticosteroids
PMN: Polymorphonuclear
PRISMA: Preferred Reporting Items for Systematic Review and Meta-analysis
PROSPERO: International Prospective Register of Systematic Reviews
RCTs: Randomized controlled trials
TGF-β1: Transforming growth factor-beta 1
Th: T helper
TNF-α: Tumor necrosis factorα
Tregs T: Regulatory T cells
