# Supplementary material for: Associations between vitamin D status and biomarkers linked with inflammation in patients with asthma: a systematic review and meta-analysis of interventional and observational studies
Source: Respir Res. 2024 Sep 19;25:344. doi: 10.1186/s12931-024-02967-z (PMC11423515; doi:10.1186/s12931-024-02967-z)
Supplement: Supplementary file 2 — Supplementary Material 2 [file 12931_2024_2967_MOESM2_ESM.docx]

**Additional file 2**: **Table S1**. Risk of bias summary using American Dietetic Association's standardized critical appraisal checklist.

| Study | Q1^1^ | Q2^2^ | Q3^3^ | Q4^4^ | Q5^5^ | Q6^6^ | Q7^7^ | Q8^8^ | Q9^9^ | Q10^10^ | Risk of bias |
| --- | --- | --- | --- | --- | --- | --- | --- | --- | --- | --- | --- |
| Interventional studies (N = 3) | | | | | | | | | | | |
| Kerley et al. (45), 2016 | Y | Y | N | Y | Y | Y | Y | N | Y | Y | Moderate |
| Shabana et al. (38), 2019 | Y | Y | N | Y | Y | Y | Y | N | Y | Y | Moderate |
| Thijs et al. (56), 2015 | Y | Y | N | Y | Y | Y | Y | N | Y | Y | Moderate |
| Cohort studies (N = 8) | | | | | | | | | | | |
| Arikoglu et al. (46), 2015 | Y | Y | Y | Y | N | Y | Y | Y | Y | Y | **Low** |
| Arikoglu et al. (57), 2017 | Y | Y | Y | Y | N | Y | Y | Y | Y | Y | **Low** |
| Bantulà et al. (58), 2022 | Y | Y | N | N | Y | Y | Y | N | Y | Y | Moderate |
| Batmaz et al. (29), 2018 | Y | Y | Y | N | N | Y | Y | Y | Y | Y | **Low** |
| Bose et al. (59), 2013 | Y | Y | N | Y | N | Y | Y | N | Y | Y | Moderate |
| Goleva et al. (30), 2012 | Y | N | Y | N | N | Y | Y | Y | Y | Y | Moderate |
| Haag et al. (44), 2018 | Y | Y | N | N | N | Y | Y | N | N | Y | Moderate |
| Hebbar et al. (60), 2014 | Y | Y | N | N | N | Y | Y | N | Y | Y | Moderate |
| Case-control studies (N = 10) | | | | | | | | | | | |
| Ahmed et al. (61), 2020 | Y | Y | N | N | Y | Y | Y | N | Y | Y | Moderate |
| Al-Athari et al. (62), 2022 | Y | N | N | N | Y | Y | Y | N | Y | Y | Moderate |
| Albanna et al. (63), 2012 | Y | N | N | N | Y | Y | Y | N | N | UC | High |
| Ehlayel et al. (42), 2011 | Y | N | N | Y | Y | Y | Y | N | Y | UC | Moderate |
| Kilic et al. (47), 2019 | Y | Y | N | N | Y | Y | Y | N | Y | Y | Moderate |
| Maalmi et al. (40), 2012 | Y | Y | N | N | Y | Y | Y | N | N | Y | Moderate |
| Mohammadzadeh et al. (64), 2020 | Y | Y | N | N | Y | Y | Y | N | Y | Y | Moderate |
| Nasiri Kalmarzi et al. (31), 2016 | Y | Y | Y | N | Y | Y | Y | Y | Y | Y | **Low** |
| Pervaiz et al. (39), 2019 | Y | Y | N | N | Y | Y | Y | N | N | UC | Moderate |
| Wawrzyniak et al. (65), 2017 | Y | Y | N | N | Y | Y | Y | N | Y | Y | Moderate |
| Cross-sectional studies (N = 50) | | | | | | | | | | | |
| Adam-Bonci et al. (48), 2020 | Y | N | N | Y | N | Y | Y | N | Y | Y | Moderate |
| Al-Attas et al. (50), 2017 | Y | Y | N | N | N | Y | Y | N | Y | Y | Moderate |
| Aldubi et al. (66), 2015 | Y | N | Y | N | N | Y | Y | Y | Y | Y | Moderate |
| Al-Thagfan et al. (33), 2021 | Y | N | N | N | N | Y | Y | N | Y | Y | Moderate |
| Alyasin et al. (67), 2011 | Y | N | N | N | N | Y | Y | N | Y | Y | Moderate |
| Alzughaibi et al. (68), 2022 | Y | N | N | N | N | Y | Y | N | Y | UC | Moderate |
| Amorim et al. (69), 2020 | Y | N | N | N | N | Y | Y | N | Y | UC | Moderate |
| Beyhan-Sagmen et al. (43), 2017 | Y | N | N | N | N | Y | Y | N | Y | Y | Moderate |
| Brehm et al. (25), 2009 | Y | Y | Y | Y | N | Y | Y | Y | Y | Y | **Low** |
| Brehm et al. (49), 2012 | Y | Y | N | Y | N | Y | Y | N | Y | UC | Moderate |
| Beigh et al. (70), 2020 | Y | N | N | N | N | Y | Y | N | Y | Y | Moderate |
| Bonanno et al. (71), 2014 | Y | N | N | N | N | Y | Y | N | N | Y | High |
| Chary et al. (72), 2016 | Y | N | N | N | N | Y | Y | N | N | UC | High |
| Checkley et al. (73), 2015 | Y | Y | Y | N | N | Y | Y | Y | Y | Y | **Low** |
| Dabbah et al. (74), 2015 | Y | N | N | N | N | Y | Y | N | Y | Y | Moderate |
| Dajic et al. (75), 2019 | Y | N | N | N | N | Y | Y | N | N | Y | High |
| Dogru et al. (76), 2014 | Y | N | N | N | N | Y | Y | N | Y | Y | Moderate |
| Ebrahimi et al. (77), 2021 | Y | Y | N | N | N | Y | Y | N | Y | UC | Moderate |
| Elnady et al. (78), 2013 | Y | N | N | N | N | Y | Y | N | N | Y | High |
| El-Said et al. (79), 2016 | Y | N | N | N | N | Y | Y | N | N | UC | High |
| Gupta et al. (41), 2014 | Y | N | N | N | N | Y | Y | N | N | Y | High |
| Hakamifard et al. (80), 2020 | Y | N | N | N | N | Y | Y | N | N | Y | High |
| Hamed et al. (81), 2016 | Y | N | N | N | N | Y | Y | N | Y | UC | Moderate |
| Havan et al. (82), 2017 | Y | N | N | N | N | Y | Y | N | Y | Y | Moderate |
| Hutchinson et al. (83), 2016 | Y | N | Y | Y | N | Y | Y | Y | Y | UC | Moderate |
| Hutchinson et al. (84), 2018 | Y | N | N | Y | N | Y | Y | N | Y | Y | Moderate |
| Janeva-Jovanovska et al. (85), 2017 | Y | N | N | N | N | Y | Y | N | Y | Y | Moderate |
| Jolliffe et al. (86), 2018 | Y | Y | Y | Y | N | Y | Y | Y | Y | UC | **Low** |
| Kalicki et al. (87), 2017 | Y | N | N | N | N | Y | Y | N | N | Y | High |
| Korn et al. (34), 2013 | Y | N | Y | N | N | Y | Y | Y | Y | Y | Moderate |
| Kuti et al. (36), 2021 | Y | N | N | N | N | Y | Y | N | Y | Y | Moderate |
| Lan et al. (88), 2014 | Y | N | N | N | N | Y | Y | N | N | Y | High |
| Li et al. (89), 2011 | Y | N | Y | N | N | Y | Y | Y | Y | Y | Moderate |
| Montero-Arias et al. (90), 2013 | Y | N | N | N | N | Y | Y | N | Y | Y | Moderate |
| Osman et al. (91), 2019 | Y | N | N | N | N | Y | Y | N | Y | Y | Moderate |
| Ozdogan et al. (92), 2017 | Y | N | N | N | N | Y | Y | N | Y | Y | Moderate |
| Ozkars et al. (93), 2019 | Y | N | N | N | N | Y | Y | N | N | Y | High |
| Ozturk Thomas et al. (94), 2019 | Y | N | N | N | N | Y | Y | N | Y | Y | Moderate |
| Pollard et al. (95), 2017 | Y | Y | Y | N | N | Y | Y | Y | Y | Y | **Low** |
| Protsiuk et al. (96), 2018 | Y | N | N | N | N | Y | Y | N | N | UC | High |
| Samaha et al. (35), 2015 | Y | N | N | N | N | Y | Y | N | Y | UC | Moderate |
| Santos et al. (97), 2018 | Y | N | N | N | N | Y | Y | N | Y | Y | Moderate |
| Searing et al. (98), 2010 | Y | N | N | N | N | Y | Y | N | Y | Y | Moderate |
| Solidoro et al. (99) 2017 | Y | N | N | N | N | Y | Y | N | Y | Y | Moderate |
| Odrowąż-Sypniewska et al. (100), 2014 | Y | Y | N | N | N | Y | Y | N | Y | UC | Moderate |
| Tamašauskienė et al. (101), 2015 | Y | N | N | N | N | Y | Y | N | N | Y | High |
| Wang et al. (37), 2018 | Y | Y | N | Y | N | Y | Y | N | Y | Y | Moderate |
| Wytrychowski et al. (102), 2020 | Y | N | N | N | N | Y | Y | N | Y | Y | Moderate |
| Yang et al. (103), 2020 | Y | N | Y | N | N | Y | Y | Y | Y | Y | Moderate |
| Yousif et al. (104), 2019 | Y | N | N | N | N | Y | Y | N | Y | Y | Moderate |
| N, No; NA, Not Applicable; UC, Unclear; Y, Yes  ^1^Q1=Question 1: Was the research question clearly stated?  ^2^Q2=Question 2: Was the selection of study subjects/patients free from bias?  ^3^Q3=Question 3: Were study groups comparable?  ^4^Q4=Question 4: Was method of handling withdrawals described?  ^5^Q5=Question 5: Was blinding used to prevent introduction of bias?  ^6^Q6=Question 6: Were intervention/therapeutic regimens/exposure factor or procedure and any Comparison(s) described in detail? Were intervening factors described?  ^7^Q7=Question 7: Were outcomes clearly defined and the measurements valid and reliable?  ^8^Q8=Question 8: Was the statistical analysis appropriate for the study design and type of outcome indicators? ^9^Q9=Question 9: Are conclusions supported by results with biases and limitations taken into consideration?  ^10^Q10=Question 10: Is bias due to study’s funding or sponsorship unlikely? | | | | | | | | | | | |
